# The Wassermann Reaction

**Published:** 1911-06

**Authors:** 


### The Wassermann Reaction.

MacRae (*New York Medical Journal,* December 31, 1910) believes as the result of a study of some fifty-four cases that it is probable that even should "606" prove harmless future medication will combine this drug with mercury in selected cases: He holds that no patient should be treated without previous Wassermann reaction, and that each case should be followed in its course and the reaction repeated in a reasonably short period; that quantitative reactions after a suitable length of time should be followed by "606" with mercury. He thinks that a certain percentage of active tertiary cases with active Wassermann reaction must have the indication for "606" treatment more clearly brought out, and that in certain cases of obscure affections of the nervous system giving a negative reaction, but with antecedent history of lues, clinical results have apparently justified the use of "606."—*Therapeutic Gazette.*

